# Hypoadiponectinemia does not enhance anxiety-like behaviour in a lean PCOS-like mouse model

**DOI:** 10.1038/s41598-026-69143-9

**Published:** 2026-09-03

**Authors:** Manisha Samad, Joakim Ek, Josefin Kataoka, Eva Lindgren, Claes Ohlsson, Ingrid Wernstedt Asterholm, Elisabet Stener-Victorin, Anna Benrick

**Affiliations:** 1 Department of Physiology, Institute of Neuroscience and Physiology, Sahlgrenska Academy, University of Gothenburg, Box 432, 405 30 Gothenburg, Sweden; 2 Department of Obstetrics and Gynaecology, Sahlgrenska University Hospital, Gothenburg, Sweden; 3 Department of Physiology and Pharmacology, Karolinska Institute, Biomedicum, Stockholm, Sweden; 4 Centre for Bone and Arthritis Research, Department of Internal Medicine and Clinical Nutrition, Institute of Medicine, Sahlgrenska Academy, University of Gothenburg, Gothenburg, Sweden; 5 Department of Biomedicine, Institute of Health Sciences, University of Skövde, Skövde, Sweden

**Keywords:** Polycystic ovary syndrome, Androgens, Adiponectin, Health-related quality of life, Anxiety, Diseases, Endocrinology, Medical research, Physiology

## Abstract

Polycystic ovary syndrome (PCOS) is characterized by reproductive and metabolic disturbances and associated with higher prevalence of anxiety and depression. Circulating adiponectin, an insulin-sensitizing adipokine, is reduced in women with PCOS, and low adiponectin has been linked to impaired mental health, particularly in females. We investigated whether low serum adiponectin is associated with impaired mental health in women with PCOS and whether adiponectin deficiency exacerbates anxiety-like behaviour in a PCOS-like mouse model. Serum adiponectin was measured in women with (n = 179) and without PCOS (n = 228), stratified by body mass index (BMI). Health-related quality of life was assessed using the short form 36 (SF-36), generating physical and mental component scores. In parallel, the prenatal androgenization (PNA) PCOS-like mouse model was combined with adiponectin-deficient mice (APNhet) to assess the impact of reduced adiponectin on anxiety-like behaviour with and without prenatal androgen exposure. Women with PCOS had lower total and high molecular weight adiponectin levels compared with controls. Adiponectin positively correlated with mental component scores in women with BMI < 30, but not in those with obesity. Free testosterone was inversely correlated with adiponectin. In mice, PNA induced anxiety-like behaviour. However, despite a 65% reduction in serum adiponectin levels in APNhet PNA mice, adiponectin deficiency did not exacerbate the anxiety-like phenotype. Unlike women with PCOS, adult PNA mice were not hyperandrogenic. These findings suggest that adiponectin is associated with mental health in non-obese women, but reduced adiponectin alone does not induce anxiety-like behaviour in the absence of hyperandrogenism. Further studies in hyperandrogenic models are needed to clarify how adiponectin and hyperandrogenism interact across developmental stages and in different BMI groups to influence vulnerability to anxiety and depression in PCOS.

Trial registration

Clinical trial.gov: NCT01319162, NCT00484705, NCT01457209, and NCT00921492.

**Supplementary Information:**

The online version contains supplementary material available at https://doi.org/10.1038/s41598-026-69143-9.

## Introduction

Polycystic ovary syndrome (PCOS) is a common endocrine disorder affecting approximately 11–13% of women of reproductive age^1^. It is a multifaceted condition with reproductive, metabolic, as well as psychological manifestations^1^. The predominant characteristics include hyperandrogenism, ovulatory dysfunction and polycystic ovarian morphology. Clinically, PCOS is often associated with hirsutism, menstrual irregularities, decreased fertility, obesity and increased risk of type 2 diabetes^1^. According to the current diagnostic criteria, PCOS is defined by the presence of at least two of the following three features: 1) clinical/biochemical hyperandrogenism, 2) ovulatory dysfunction, and 3) polycystic ovaries on ultrasound or elevated anti-müllerian hormone (AMH) levels^1^.

Beyond its reproductive and metabolic consequences, PCOS is strongly associated with impaired mental health^2^. Screening for anxiety and depression is therefore recommended at the time of diagnosis^3^. However, many patients report that psychological issues are under-recognized, and report dissatisfaction with the support and counselling received^4^. While much of the research on PCOS has focused on its reproductive and metabolic features, women with PCOS also report significantly lower mental health–related quality of life (HRQoL)^2,5^. They also experience a high burden of psychological symptoms, with studies reporting depressive symptoms in approximately 30–40% of women and anxiety symptoms in roughly 30–75%, depending on the population studied and the assessment method used^6–8^. This could be due to the social stigma of PCOS-symptoms such as obesity, infertility, acne, and hirsutism, along with the chronic, complex, and frustrating nature of PCOS, contributing to depression and anxiety symptoms^9^. In addition, anxiety and depressive symptoms have been linked to hyperandrogenism and insulin resistance, suggesting underlying biological contributors beyond psychosocial stressors^10^. Although PCOS is a heritable disorder, currently identified genetic variants explain only approximately 10% of the estimated 70% heritability, indicating that PCOS develops due to a combination of genetic and environmental factors^11^. Moreover, summary statistics from genome-wide association studies suggest no shared genetic basis or causal relationship of PCOS with psychiatric disorders including depression, anxiety, schizophrenia and bipolar disorder, suggesting that the association is likely mediated by BMI^12^.

Both clinical and preclinical studies highlight the role of excess androgens in the development of PCOS^2,13,14^. In pregnant women with PCOS, elevated androgen levels persist throughout gestation, and androgens can cross and negatively affect the placenta structure and function, potentially influencing foetal development and the development of PCOS symptoms^15–17^. Prenatal androgen exposure has been shown in both human and animal studies to affect cognitive and behavioural outcomes in female offspring^17,18^. We and others have demonstrated that prenatal androgen exposure leads to the development of anxiety-like behaviour in female rodents^13,17^. Consistent with these findings, children of women with PCOS are at increased risk of neuropsychiatric disorders, including anxiety, depression, ADHD, and autism spectrum disorder^18^. Using Swedish population-based health registers, we have reported an 78% increased risk of anxiety diagnosis among daughters of women with PCOS ^17^. Together, these findings suggest that maternal androgen excess may contribute to long-term neuropsychiatric vulnerability in female offspring.

Another potential mediator linking PCOS and impaired mental health is reduced adiponectin levels. Adiponectin is an adipocyte-produced insulin-sensitizing hormone with important metabolic properties^19^. Emerging evidence suggests sex-specific interactions between severe mental disorders and circulating levels of testosterone, sex hormone–binding globulin (SHBG), and adiponectin, with women exhibiting higher testosterone and lower adiponectin levels in association with psychiatric conditions, pointing to a potential sex-dependent pathophysiology^20^. In women with PCOS, adiponectin levels are lower and correlates with insulin resistance^21,22^. A meta-analysis further demonstrated an inverse association between circulating adiponectin and psychiatric disorders, including anxiety, mood, and trauma-related disorders^23^. Notably, the association with depressive disorders was stronger in females^20^. Together, these findings suggest that adiponectin may represent a biological link between metabolic dysfunction, hyperandrogenism, and mental health vulnerability in women.

**Fig. 1:**
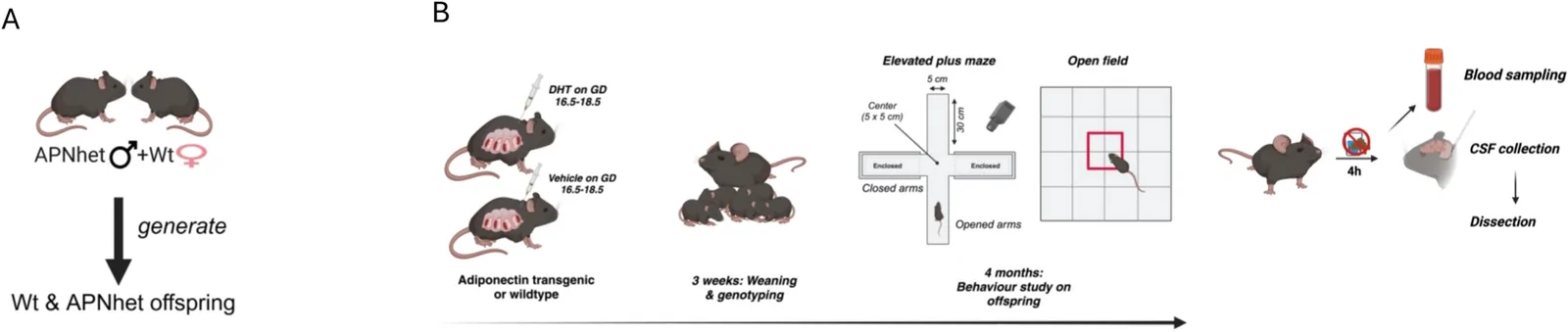
Study design. **(A)** Schematic representation of the breeding scheme generating wildtype (wt) & adiponectin heterozygous (APNhet) littermates and **(B)** a description of the overall study plan where the effects of adiponectin deficient with and without prenatal androgenization (PNA) on anxiety-like behaviour were studied using elevated plus maze and open field followed by dissection. Dihydrotestosterone; DHT (created with BioRender.com).

However, the mechanisms by which adiponectin and androgens influence mental health remain poorly understood. We therefore investigated whether low serum adiponectin is associated with impaired mental health symptoms in women with and without PCOS, and whether reduced adiponectin exacerbates anxiety-like behaviour in a PCOS-like mouse model. To examine the interaction between adiponectin and anxiety mechanistically, the prenatal androgenization (PNA) model was combined with adiponectin-deficient mice (Fig. 1). The PNA model develops anxiety-like behaviour and is characterized by PCOS-like reproductive and metabolic disturbances^17,24^.

## Results

### Clinical cohort

Detailed anthropometric data and mental health scores from the four studies have been previously published^25–29^. Normal-weight and overweight women with PCOS in this study have been shown to have more symptoms of depression and lower mental component score (MCS) compared with controls, and normal-weight women with PCOS have been shown to have more symptoms of anxiety^29^. Based on this finding, we divided the cohort into BMI < 30 kg/m^2^ and BMI ≥ 30 kg/m^2^. Secondary analyses of PCOS-characteristic variables and mental health scores divided into BMI < 30 kg/m^2^ and BMI ≥ 30 kg/m^2^ are presented in Table 1. Women with PCOS have higher Ferriman-Gallwey (FG)-score, total testosterone, free testosterone and free androgen index (FAI) and lower SHBG, weight, BMI, and fasting insulin did not differ between groups (Table 1). In those with BMI < 30 kg/m^2^, symptoms of depression and anxiety was higher and MCS was lower in women with PCOS compared to controls (Table 1).

**Table 1:** Metabolic and psychological characteristics of women with PCOS and controls stratified by BMI (< 30 and ≥ 30 kg/m^2^).

Variable	BMI < 30 Controls (n = 37)	BMI < 30 PCOS (n = 76)	P-value	BMI ≥ 30 Controls (n = 191)	BMI ≥ 30 PCOS (n = 103)	*P*-value	Adj *P*-value
Age (y)	27.7	28.8	0.194	37.6	32.5	< 0.001	
Weight (kg)	69.8	67.1	0.287	110.3	105.7	0.015	0.059
BMI (kg/m^2^)	24.9	23.8	0.132	39.4	38.2	0.032	0.081
FG score	0.4	10.6	< 0.001	3.2	12.3	< 0.001	< 0.001
SHBG (nmol/L)	67.3	47.0	< 0.001	33.2	27.2	< 0.001	< 0.001
Total testosterone (nmol/L)	0.78	1.34	< 0.001	1.11	1.62	< 0.001	< 0.001
Free testosterone (nmol/L)	0.0096	0.0213	< 0.001	0.0209	0.0331	< 0.001	< 0.001
FAI	1.47	3.73	< 0.001	3.95	6.97	< 0.001	< 0.001
F-insulin (mU/L)	6.68	7.32	0.194	17.61	19.19	0.028	0.102
CPRS-S-A Anxiety	9.19	14.40	< 0.001	16.60	16.00	0.689	0.786
CPRS-S-A Depression	6.76	13.20	< 0.001	13.60	13.40	0.891	0.983
SF-36 MCS	46.3	32.3	< 0.001	38.1	36.6	0.335	0.551

Data are shown for controls and women with PCOS within each BMI category. Comparisons between controls and women with PCOS were performed using Mann–Whitney *U* test. Adjusted *P* values were obtained by ANCOVA with adjustment for age. Abbreviations: PCOS, polycystic ovary syndrome; FG score, Ferriman–Gallwey score; SHBG, sex hormone-binding globulin; FAI, free androgen index; F-insulin, fasting insulin; CPRS-S-A, Comprehensive Psychopathological Rating Scale (CPRS) Self-rating Scale for Affective Syndromes; SF-36 MCS, Short Form 36 Mental Component Summary.

**Fig. 2:**
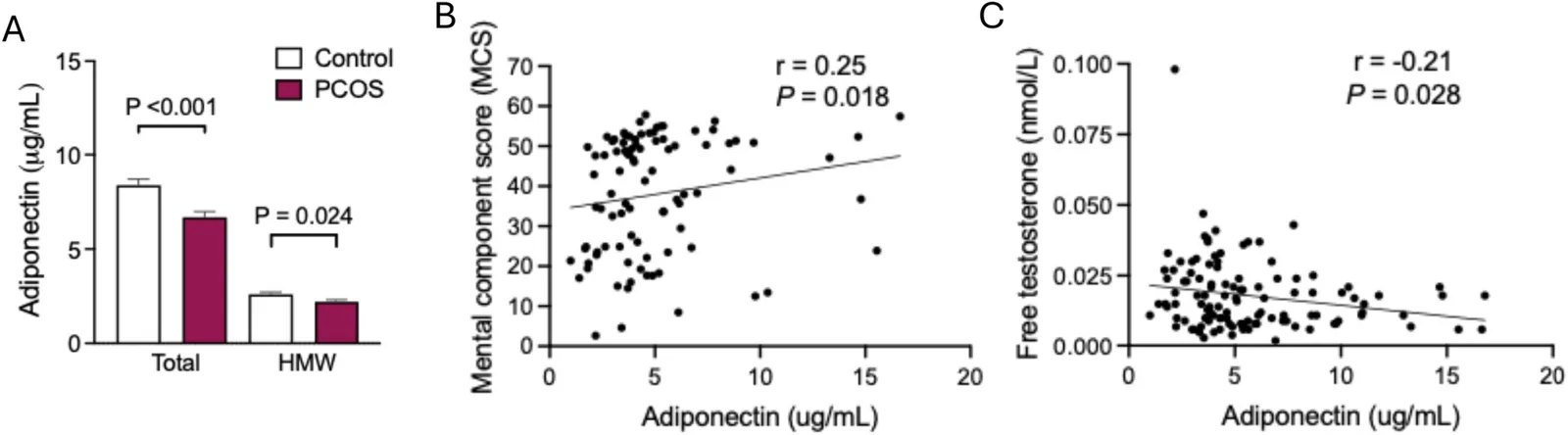
Clinical cohort. **(A)** Total and high-molecular weight (HMW) adiponectin in women with PCOS (n = 179) and controls (n = 228), **(B)** Correlation between serum adiponectin and mental component summary (MCS) score and **(C)** between serum adiponectin and free testosterone in women with BMI < 30 (n = 113). Mann–Whitney U-test was used to compare women with and without PCOS. Correlations were analyzed using Spearman’s rank correlation test. Data are expressed as mean ± SEM.

Total and high-molecular-weight (HMW) adiponectin levels were lower in women with PCOS compared to controls (Fig. 2A), with no difference in the HMW/total adiponectin ratio (0.332 ± 0.011 vs 0.338 ± 0.011,* P* = 0.51).

There were no differences in symptoms of anxiety, depression, or MSC in women with obesity and severe obesity^29^, therefore we performed correlation analyses including only women with BMI < 30 kg/m^2^ to determine if adiponectin correlates with mental health scores. Higher total adiponectin showed a weak correlation with MCS (r = 0.25, Fig. 2B**)**, while there was no correlation between adiponectin and anxiety (r_spearman (s)_ = -0.052,* P* = 0.58) or depression (r_s_ = -0.090,* P* = 0.34*)*. Testosterone inhibits adiponectin expression and secretion from adipocytes in vitro^30,31^. In line with this, increased free testosterone (r = -0.21, Fig. 2C**)** and free androgen index (FAI) had a weak correlation with lower adiponectin *(*r_s_ = -0.20,* P* = 0.037).

### Behaviour characteristics

**Fig. 3:**
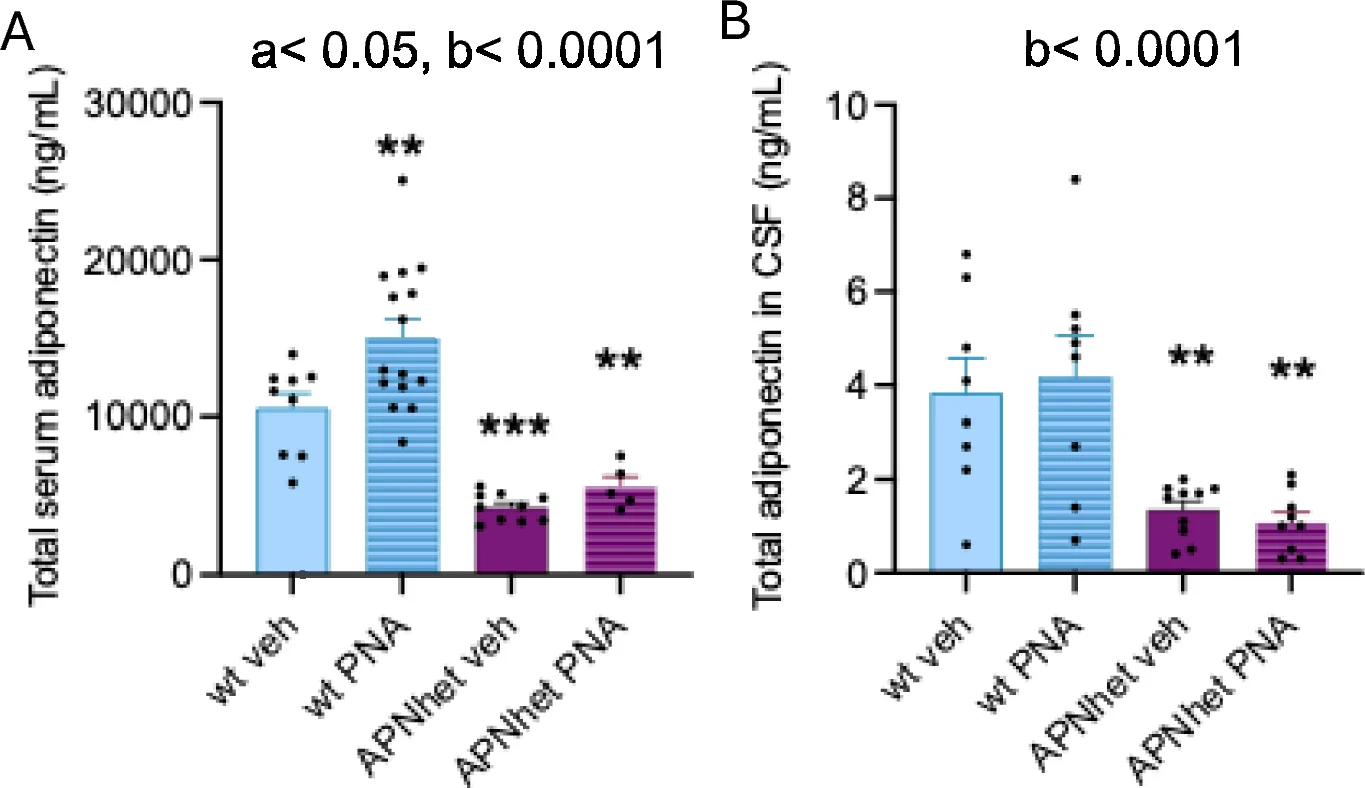
Adiponectin levels in (**A**) serum and (**B)** CSF in 4-month-old wild-type (wt) and adiponectin heterozygous (APNhet) female mice with and without prenatal androgenization (PNA) (wt veh n = 9 and 8, wt PNA n = 15 and 8, APNhet veh n = 10 and 10, and APNhet PNA n = 5 and 9, in serum and CSF, respectively). A two-way ANOVA was used to test the independent main effects of PNA and genotype on serum and CSF adiponectin, and any interaction between them, a; main effect of PNA, b; main effect of APNhet. Data are expressed as mean ± SEM and **P < *0.01,* ***P < *0.001* vs wt veh.

An adiponectin-deficient mouse model was used to move the above finding from correlation to causality. Adiponectin-deficient mice were used to determine whether lower adiponectin enhances anxiety like-behaviour in prenatal androgenized mice. APNhet, an adiponectin deficient model, showed a 65% decrease in serum adiponectin compared to wild type (wt) **(**Fig. 3A**)**. A similar effect was seen in cerebrospinal fluid (CSF), confirming that central adiponectin was also decreased in APNhet mice **(**Fig. 3B**)**. There was a strong correlation between serum and CSF levels (*r* = *0.65, P* < 0.0001).

**Fig. 4:**
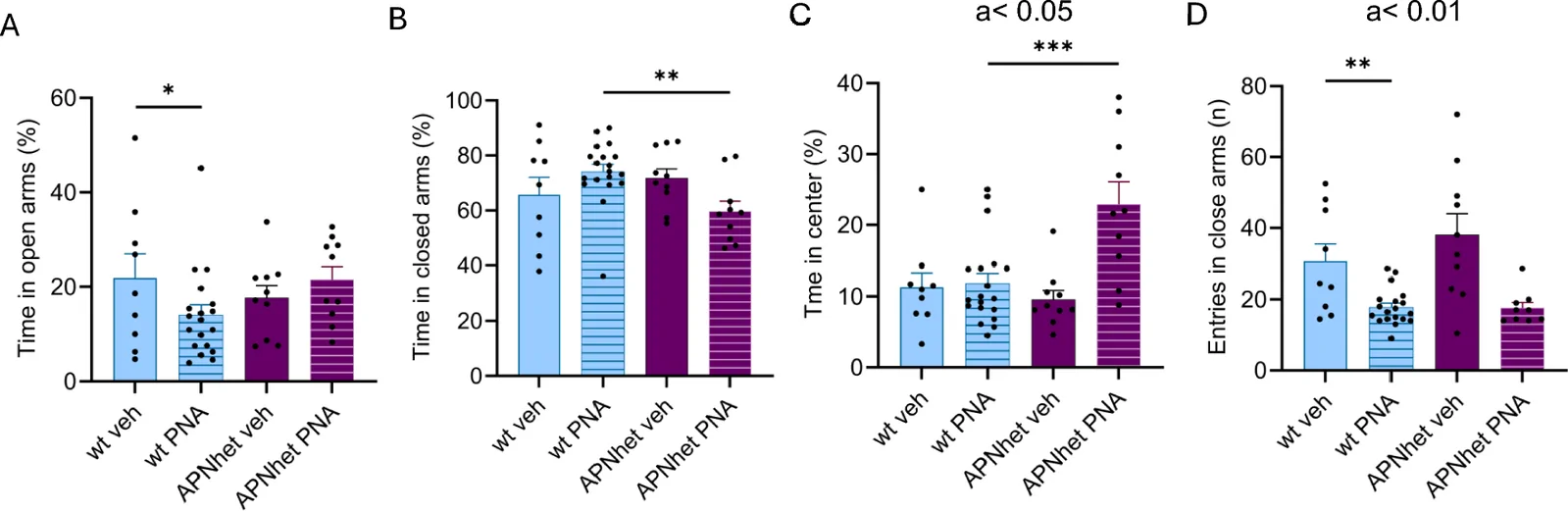
Behaviour characteristics in elevated plus maze (EPM). **(A)** Time spent in open arms, **(B)** Time spent in closed arms, **(C)** Time spent in centre, and **(D)** number of entries into closed arms in 4-month-old wild-type (wt) and adiponectin heterozygous (APNhet) female mice with and without prenatal androgenization (PNA) (wt veh n = 9, wt PNA n = 19, APNhet veh n = 10, and APNhet PNA n = 10). The effect of PNA in wt mice was analysed by an unpaired Student’s t-test, and wt PNA mice were compared to APNhet PNA to determine if there was an additive effect of adiponectin deficiency. Data are expressed as mean ± SEM and *P < 0.05, **P < *0.01,* ***P < *0.001*. A two-way ANOVA was used to test the independent main effects of PNA and genotype on behavioural measures, and any interaction between them, a; main effect of PNA.

As expected, wt PNA mice spent less time in the open arms and tended to spend more time in the closed arms, indicating anxiety-like behaviour compared to the wt vehicle group (Fig. 4A, B). APNhet PNA mice spent more time in the centre compared to wt PNA** (**Fig. 4C**)**. The number of entries into the closed arms was also lower in wt PNA offspring, indicating that the animals preferred to stay in the closed arms **(**Fig. 4D**).** APNhet mice exhibited normal locomotor activity and there was no evident difference in behaviour in unchallenged or PNA mice compared to wt, except for number of entries into the closed arms, which was lower in APNhet PNA **(**Fig. 4A-D**)**. A two-way ANOVA showed a main effect of PNA on fewer entries in closed arms and more time in centre of the EPM (Fig. 4 C, D). There was no difference in the time spent in the centre zone, the number of entries into the centre zone, or the distance moved in the open field between groups (Supplement Table 2). There was no interaction effect between genotype and PNA for any of the behavioural outcomes (Supplement Table 3).

### Metabolic and reproductive characteristics

There was no difference in body weight, inguinal white adipose tissue, gonadal white adipose tissue, and liver weight between the groups **(**Table 2**)**. There was no effect of PNA on fasting glucose, but APNhet exhibited lower fasting glucose levels than wt (Table 2). In contrast, fasting insulin levels were lower in wt PNA than in wt veh, and there was a main effect of PNA on lowering blood glucose (Table 2**).**

**Table 2:** Metabolic characteristics of 4-month-old wild-type (wt) and adiponectin heterozygous (APNhet) female mice with and without prenatal androgenization (PNA).

Variable	wt veh	wt PNA	APNhet veh	APNhet PNA	two-way ANOVA
Body weight (g)	27.7 ± 0.8	27.8 ± 0.7	27.1 ± 0.9	27.5 ± 1.2	ns
IWAT (g)	0.93 ± 0.11	0.98 ± 0.07	0.78 ± 0.10	0.81 ± 0.11	ns
GWAT (g)	1.16 ± 0.11	1.18 ± 0.09	0.91 ± 0.14	1.19 ± 0.16	ns
Liver (g)	1.04 ± 0.04	0.98 ± 0.03	0.98 ± 0.06	0.98 ± 0.03	ns
Ovaries (mg)	12 ± 0.7	13 ± 1.0	14 ± 2.6	12 ± 1.3	ns
Glucose (mmol/L)	8.7 ± 0.36	8.1 ± 0.17	7.6 ± 0.27	7.5 ± 0.28	b < 0.01
Insulin (ng/mL)	1.62 ± 0.19	1.12 ± 0.11*	1.27 ± 0.16	0.95 ± 0.19	a > 0.05

A Mann–Whitney *U*-test was used to compare wt vehicle versus wt PNA to establish the effect of PNA, and wt PNA versus APNhet PNA to determine if there was an additive effect of adiponectin deficiency (wt veh n = 9, wt PNA n = 19, APNhet veh n = 10, and APNhet PNA n = 10). A two-way ANOVA was used to test the independent main effects of PNA and genotype and any interaction between them; a; main effect of PNA, b; main effect of APNhet. IWAT; inguinal adipose tissue; GWAT, gonadal adipose tissue. Data are expressed as mean ± SEM and *P < *0.05* vs wt veh, ns; non-significant.

**Fig. 5:**
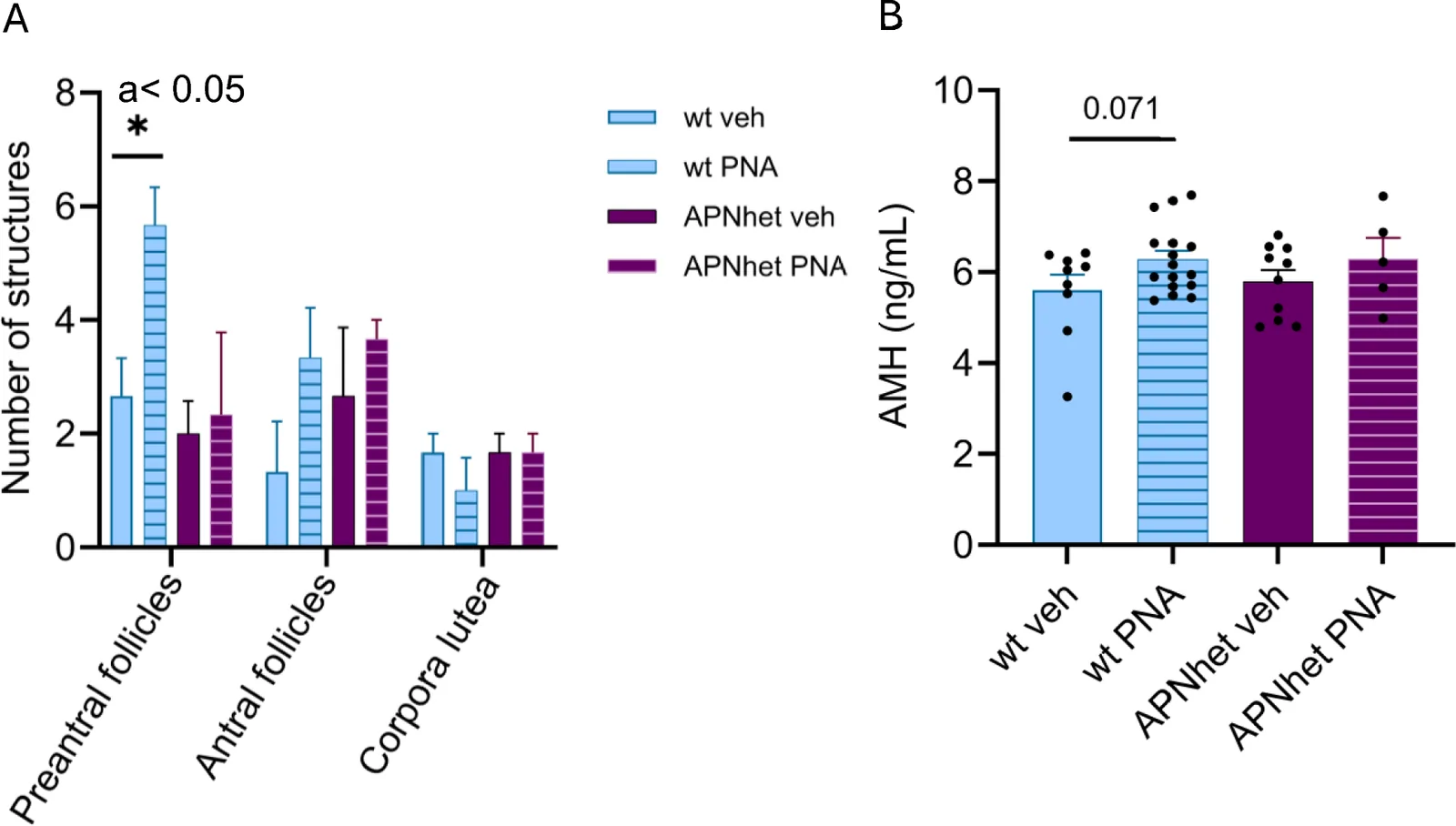
Ovarian morphology. **(A)** Quantitative analyses of the mean number of preantral, antral and corpora lutea (CL) (n = 4/group ), and **(B)** serum anti-müllerian hormone (AMH) levels in 4-month-old wild-type (wt) and adiponectin heterozygous (APNhet) offsprings with and without prenatal androgenization (PNA) (wt veh n = 9, wt PNA n = 16, APNhet veh n = 10, and APNhet PNA n = 5). The effect of PNA in wt mice was analysed by an unpaired Student’s t-test, and wt PNA mice were compared to APNhet PNA to determine if there was an additive effect of adiponectin deficiency. Data are expressed as mean ± SEM and *P < *0.05*. A two-way ANOVA was used to test the independent main effects of PNA and genotype on reproductive outcomes, and any interaction between them, a; main effect of PNA.

PNA wt mice had an increased number of preantral follicles and there was a main effect of PNA on increased number of preantral follicles, and a trend toward more antral follicles (main effect of PNA p = 0.12) **(**Fig. 5A**).** An increase in antral follicles is often associated with polycystic ovarian morphology and anovulation in women with PCOS. However, no differences were observed in ovary weight or the number of corpora lutea **(**Table 2**, **Fig. 5A**)**. We measured AMH in the serum as a marker for polycystic ovary morphology. In wt mice, PNA exposure tended to increase AMH concentrations relative to wt veh; however, this difference did not reach statistical significance *(P* = 0.071) **(**Fig. 5B**)**. Sex hormone levels did not differ between groups (Table 3). Serum testosterone and DHT were below the lowest level of quantification in many mice, especially in the PNA groups, resulting in many missing values. This contrasts with the clinical cohort, where women with PCOS are hyperandrogenic.

**Table 3:** Serum concentrations of sex hormones in 4-month-old wild-type (wt) and adiponectin heterozygous (APNhet) female mice with and without prenatal androgenization (PNA).

Variable	wt veh	wt PNA	APNhet veh	APNhet PNA	Fisher’s exact test, *P*-value
Androstenedione (pg/mL)	48.2 ± 4.4 n = 10	46.1 ± 4.2 n = 10	50.0 ± 10.3 n = 10	51.9 ± 4.4 n = 10	na
Progesterone (pg/mL)	5351 ± 1356 n = 10	2627 ± 673 n = 10	4808 ± 1779 n = 10	3676 ± 2267 n = 10	na
DHT (pg/mL)	9.7 ± 1.7 n = 3	– n = 0	7.1 ± 2.6 n = 6	6.5 ± 0.0 n = 1	0.008
Estradiol (pg/mL)	3.18 ± 1.10 n = 9	1.68 ± 0.46 n = 8	3.78 ± 1.22 n = 10	3.04 ± 0.74 n = 9	0.61
Testosterone (pg/mL)	17.2 ± 3.7 n = 8	11.3 ± 1.2 n = 3	21.9 ± 5.5 n = 6	27.5 ± 5.8 n = 8	0.33

A Mann–Whitney *U*-test was used to compare wt vehicle versus wt PNA to establish the effect of PNA, and wt PNA versus APNhet PNA to determine if there was an additive effect of adiponectin deficiency (all non-significant). The presence of sex hormones above the lowest level of quantification in vehicle vs. PNA exposed mice was analyzed by Fisher’s exact test. n = 10/group, if n < 10, excluded samples were below the lowest level of quantification. Data are presented as mean ± SEM.

## Discussion

Women with PCOS report higher levels of anxiety, depressive symptoms, and lower health related quality of life compared with women without PCOS^8,32,33^. In the present study, lower serum adiponectin was weakly associated with poorer mental health scores, suggesting that reduced adiponectin may contribute to the increased psychological burden observed in PCOS. We also found a weak inverse correlation between adiponectin and free testosterone, consistent with previous evidence that androgens suppress adiponectin secretion from adipocytes^30,31^. Although both testosterone and adiponectin are well known for their peripheral metabolic effects, they can also cross the blood–brain barrier and are detectable in cerebrospinal fluid, indicating potential central actions^34,35^. To disentangle the relationship between CSF adiponectin and mental health outcomes, we used a genetically modified mouse model with low adiponectin levels^36^. Contrary to our hypothesis, reduced adiponectin did not enhance androgen-induced anxiety-like behaviour, and no significant interaction between genotype and PNA exposure was detected. However, the study may have lacked sufficient statistical power to detect moderate interaction effects. These findings suggest that, although adiponectin is weakly associated with mental health measures in women with PCOS, reduced adiponectin alone may not independently exacerbate androgen-driven anxiety-related behaviour, at least under the experimental conditions used here.

Symptoms of depression and anxiety results from complex interactions between brain circuits and neurotransmitter systems^37^. For testosterone and adiponectin to influence these behaviours, they likely need to access and act within the central nervous system. According to the Developmental Origins of Health and Disease (DOHaD) hypothesis, conditions during foetal life can program long-term disease risk in adulthood^38^. Daughters of women with PCOS are exposed in utero to higher androgen levels^15^ , which may constitute an initial “hit” affecting brain development and rewiring of brain circuits. As these daughters later develop PCOS themselves^39,40^, persistent hyperandrogenism and reduced adiponectin during reproductive life may represent a second exposure, potentially reinforcing these early changes^41^. The PNA mouse model does not fully replicate this endocrine milieu. Wild-type dams were used for breeding, exposing foetuses to elevated androgens but normal maternal adiponectin. Because maternal adiponectin does not cross the placenta^42^, and foetal adiponectin production in mice begins late in gestation (around embryonic day 17) at relatively low levels^43^, prenatal programming in this model is likely driven primarily by androgen exposure. This differs from humans, where foetal adiponectin production begins in the second trimester, coinciding with adipose tissue development^44^.

Prenatal androgen exposure is linked to PCOS-like phenotypes in the offspring, illustrating an epigenetic programming of somatic tissues^45,46^, and likely also on the brain, but does not result into hyperandrogenism in adult mice. In adulthood, PNA APNhet offspring display reduced adiponectin but unchanged circulating androgens. Although adiponectin levels were threefold lower in the cerebrospinal fluid of APNhet mice, this reduction did not exacerbate PNA-induced anxiety-like behaviour. While the adipose tissue–brain axis has emerged as a potential modulator of anxiety, and adiponectin has been suggested to exert anxiolytic effects in males^47,48^, our findings do not support a direct anxiogenic effect of reduced adiponectin in female mice. A possible explanation for the absence of a clear anxiety-like phenotype in APNhet female offspring is that most mechanistic evidence linking adiponectin to anxiety-related behaviour comes from male mice. These studies show that adiponectin signalling engages anxiety-relevant pathways, including adiponectin receptor 1-mediated inhibition of ventral tegmental area dopamine neurons, producing anxiolytic-like effects, whereas adiponectin haploinsufficiency increases dopamine neuron activity and anxiety-like behaviour^47^. Adiponectin signalling has also been implicated in hippocampal function, as treatment with the adiponectin agonist AdipoRon reduced anxiety- and depression-like behaviour, although independently of synaptic plasticity^49^. Importantly, the limited evidence including both sexes suggests that adiponectin signalling is sexually dimorphic. Li et al. demonstrated that adiponectin receptor 1 signalling in serotonergic neurons regulates mood-related behaviour in a sex-dependent manner, with loss of serotonergic markers in males but not females despite some behavioural effects in both sexes^50^. Together, these findings suggest that adiponectin modulates affective behaviour through multiple central mechanisms in male mice, but whether these operate similarly in females remains unknown.

Species differences in foetal adiponectin dynamics, as well as the absence of adult hyperandrogenism in the PNA model, may contribute to the discrepancy between the human cohort and the experimental data. The lack of a more pronounced anxiety-like phenotype in adiponectin-deficient mice may therefore reflect the requirement for concurrent hyperandrogenism to unmask such effects. We cannot rule out a potential interaction between low adiponectin levels and hyperandrogenism during the postpubertal period. Further studies should address this possibility using a mouse model that combines PNA with chronic peripubertal androgen exposure.

Higher insulin levels have been associated with increased symptoms of anxiety and depression in women, both with and without PCOS^29^. Studies have shown that insulin resistance in the brain can disrupt dopamine signalling and contribute to mood disorders^51^. As insulin resistance and impaired glucose homeostasis can influence brain function through altered insulin signalling in the CNS, low-grade inflammation, and changes in neurotransmitter systems^52,53^, we used a lean mouse model to minimize the confounding effect of metabolic dysfunction. The PNA model did not exhibit insulin resistance, on the contrary, wt PNA mice showed unaltered glucose and lower fasting insulin levels, which contrasts with previous studies reporting androgen-induced glucose intolerance^54^. Notably, the APNhet mice displayed lower fasting glucose, indicating improved glucose homeostasis. We hypothesize that this unexpected finding may be explained by increased uncoupling protein-1 (UCP-1) expression in brown adipose tissue and a trend toward increased UCP-1 in white adipose tissue as this activation has been linked to enhanced insulin sensitivity and glucose utilization in mice^55,56^ . Consequently, the relatively favourable metabolic profile of APNhet mice may have attenuated neurobiological changes typically associated with metabolic dysfunction, thereby masking potential anxiogenic effects of hypoadiponectinemia.

Higher BMI has been associated with increased symptoms of anxiety and depression in women with PCOS^57^. However, in our cohort, this association was observed only among women with BMI < 30. In women with obesity, anxiety and depression scores were comparable between those with and without PCOS^29^, suggesting that obesity may override the additional impact of hyperandrogenism on mental health. Consistent with this, the positive correlation between adiponectin and mental component scores was present only in women with BMI < 30. In the highest BMI group, adiponectin levels did not differ between women with and without PCOS, further supporting the notion that obesity per se exerts a stronger influence on metabolic and psychological health than PCOS status alone.

The clinical cohort was assembled from four different studies conducted over an 11-year period. Strengths of the cohort include its relatively large sample size, the broad range of BMI categories represented, recruitment of participants from the same geographic region, analysis of all blood samples at the accredited laboratory of Sahlgrenska University Hospital, and the use of the same validated questionnaires across all studies. The study also has limitations. Participants were recruited through advertisements and from a hospital setting, therefore, the sample is not representative of an unselected population. In addition, PCOS was diagnosed using either the NIH or the Rotterdam criteria, introducing heterogeneity in PCOS phenotypes across the cohort, which may have affected the observed associations.

Clinically, these findings suggest that BMI should be carefully considered when evaluating mental health in women with PCOS. In women with BMI < 30, hyperandrogenism and adipokine dysregulation may play a more prominent role in mood symptoms. Future interventional studies should examine whether metabolically focused treatments can alleviate mood symptoms and whether treatment responses differ according to BMI and endocrine phenotype.

In conclusion, serum adiponectin is associated with mental component scores in women, suggesting a link between adipokine regulation and psychological well-being. However, reduced adiponectin alone, without hyperandrogenism and hyperinsulinemia, did not induce anxiety-like behaviour in our mouse model. The differing patterns observed across BMI categories, as well as between the human cohort and experimental data, underscore the complexity of the mechanisms underlying mental health disturbances in PCOS. Our findings point to a multifactorial interplay between adiponectin, testosterone, and obesity in shaping mental health outcomes. Further studies using hyperandrogenic models are needed to clarify how these factors interact across developmental stages and metabolic contexts to influence vulnerability to anxiety and depression in PCOS.

## Material and methods

### Clinical cohort

Data from four studies conducted in Sweden have been used for this study^25–28^ resulting in a total 407 women aged 18–38 years (PCOS n = 179, non-PCOS n = 228). A summary of each study is described in Supplemental Table 1. The studies included women in a wide range of BMIs, recruited from the community or an obesity unit between 2005–2016. The patients were screened for PCOS with the Rotterdam-criteria (two out of three criteria; hyperandrogenism, irregular cycles, and/or polycystic ovary morphology) except for the severely obese cohort^28^ where the National Institute of Health (NIH) criteria (hyperandogenism and irregular cycles) was used, as ultrasound was not a successful method to quantify polycystic ovary morphology. Ethical approval for the four studies was received from the Ethics Committee, University of Gothenburg, Gothenburg, Sweden. All studies were registered at clinicaltrials.gov; NCT01319162, NCT00484705, NCT01457209, and NCT00921492. The included studies were performed in accordance with the Declaration of Helsinki, and all participants gave their oral and written informed consent to participate in the study.

### Data collection

Data on symptoms of anxiety and depression HRQoL were assessed from the questionnaires Comprehensive Psychopathological Rating Scale (CPRS) Self-rating Scale for Affective Syndromes (CPRS-S-A), and Short Form 36 (SF-36). To assess symptoms of anxiety and depression we used the CPRS-S-A. It is a validated and well-established instrument used both in research and clinical routine psychiatric care for defining symptoms of anxiety and depression and for evaluation of treatment. From CPRS-S-A, the subscales Brief Scale for Anxiety (BSA) to assess symptoms of anxiety, and Montgomery Åsberg Depression Rating Scale (MADRS-S) to assess symptoms of depression, are extracted^58,59^. The scales consist of nine domains each, of which two are present in both scales. All domains are rated on a six-point scale: 0 represents an absence of symptoms; 2 represents a potentially pathological deviation; 4 represents a pathological condition and 6, an extremely pathological condition. Domain ratings are summarized, and a maximum value of 54 for each scale is calculated. A cut-off value of > 11 in the BSA-S and the MADRAS-S scales was used to set a reference value for clinically relevant anxiety and depression.

HRQoL is a way of describing personal health status, including physical and mental health. To assess HRQoL, SF-36 questionnaire was used. It is generic and designed to capture a person’s perception of how his or her health status has interfered with physical, psychological, and social functioning for the past four weeks, and is a validated instrument widely used^60^. It is divided into eight domains: physical functioning, physical role limitation, bodily pain, general health perception, emotional role limitation, vitality, mental health, and social functioning. In each domain a score of 0–100 is calculated, where 0 represents the worst possible health quality and 100 represents the best possible health quality. Summary scores were computed to reflect the physical component score (PCS) and MCS, scoring on a scale of 0–100.

BMI was calculated using weight in kilos, divided with squared height in meters. Hirsutism was assessed with Ferriman-Gallwey (FG)-score. Serum samples were collected in the morning from subjects fasted overnight. Total and HMW adiponectin levels were measured by ELISA (80-ADPHU-E01, Alpco, Salem, NH, USA). Serum samples were thawed, diluted and treated according to the manufacturer’s instructions. The absorbance was measured using a plate reader (SpectraMax iD3, Molecular Devices, San Jose, CA, USA). Testosterone, SHGB, and insulin were analyzed by ECLIA (CBAS 8000 Roche Diagnostics Scandinavia AB, Sweden) at the ISO-accredited laboratory at Sahlgrenska University Hospital, Gothenburg, Sweden. Free testosterone was calculated using total testosterone and SHBG according to Vermeulen et al.^61^. FAI was calculated as (total testosterone (nmol/L) / SHBG (nmol/L) × 100.

### Animal Model

A mouse model with decreased adiponectin levels was used to study the effect of adiponectin, with and without PNA. Adiponectin knockout mice were originally generated in Philipp E. Scherer’s laboratory^36^. APNhet mice were generated by crossing C57BL/6 J wild-type mice with adiponectin knockout mice on a C57BL/6 J background^36^. The mice used in this study were bred and maintained at the Laboratory of Experimental Biomedicine, University of Gothenburg. All the animals had ad libitum access to food and water and were maintained under standard housing conditions (12-h light/dark cycle environment, and fixed temperature and humidity) provided by the Laboratory of Experimental Biomedicine, University of Gothenburg. The study was approved by the Ethics Committee of the University of Gothenburg (3229/20 and 6784/25) in accordance with the ARRIVE guidelines, the European Union guidelines for the care and use of laboratory animals (2010/63/EU), and the Swedish Board of Agriculture´s regulations and general advice of laboratory animals (L150).

### Experimental design

APNhet males were mated with wt females and gave birth to both wt and APNhet offspring allowing us to study littermates (see breeding scheme and study outline in Fig. 1). Before mating, vaginal smears were performed to determine the oestrous cyclicity of the female mice^62^. The females in oestrus phase were allowed to mate with APNhet males overnight. The weight was monitored to confirm for pregnancy associated weight gain. At GD 16.5 to 18.5, vehicle or 250 μg 5α-Androstan-17β-ol-3-one (dihydrotestosterone (DHT), A8380, Sigma-Aldrich, St. Louis, USA) dissolved in 2.5 μl benzyl benzoate (B6630, Sigma-Aldrich) and 47.5 μl sesame oil (S3547, Sigma-Aldrich) was injected subcutaneously in the interscapular area. The breeding resulted in 48 mice divided into 4 groups: wt or APNhet offspring from vehicle injected wt dams (wt veh, n = 9, APNhet veh, n = 10) or DHT-injections during gestation to induced PNA (wt PNA, n = 19, APNhet PNA, n = 10). Female offspring were weaned at 3 weeks of age and genotyped as previously described^63^. Ear biopsies were incubated with DirectPCR lysis buffer (Viagen #102-T) and Proteinase K solution (Invitrogen Direct PCR #25,530–049, 0.2 mg/mL) at 55^0^C overnight and then at 80^0^C for 45 min to inactivate the enzyme. Lysate was centrifuged at 14,000 × *g* for 3 min. Two microliters of each sample was mixed with 18 µL master mix containing PCR Supermix (ThermoFisher, #10,572,014) with the following primers: wt-forward, 5′-TTCAATTCCAGCACCCACAGTAA -3′; wt-reverse, 5′-GGACCCCTGAACTTGCTTCAC-3′; and APNhet-forward 5′-GTAGCCGGATCAAGCGTATG-3′; and APNhet-reverse, 5′ATGAACTCCAGGACGAGGCA-3′. The PCR was performed with an enzyme activation step, 5 min at 95 °C, then 30 s at 95 °C, 30 s at 58 °C, 1 min at 72 °C for 35 cycles. The resulting PCR products were run separately on a 2% agarose gel, and the mice were genotyped by the presence of a 350-bp amplicon for the APNhet allele and a 350-bp amplicon for the wild type allele.

### Behavioural tests

Behavioural tests were carried out at 4 months of age. Before starting the experiment, all mice were allowed to acclimate for one hour in the room where the experiments were conducted. Anxiety-like behaviour was evaluated using an elevated plus maze (EPM) comprised of two open and two closed arms (45 × 10 cm) and a platform in the middle (10 × 10 cm), elevated 50 cm above the ground. The mouse was placed in the junction and allowed to move freely for five minutes, which was recorded using a camera mounted above the maze. The maze was placed into a dimly lit room so that light intensity reached 10–15 lx in the closed arms and 50–55 lx in the open arms. Movement was immediately tracked and prepared for further analysis using EthoVision 13 XT. Data were evaluated for time spent in the closed and open arms of the maze, time spent in the middle, number of entries in the arms, and distance travelled. This test is based on the innate aversion of mice against bright and open spaces and mice are more anxious when they spend less time in the open arms of the apparatus.

As an additional measurement of anxiety-like behaviour, mice were placed in a brightly lit open filed (OF, 100 × 100x40cm) and allowed to freely explore for 15 min. Levels of anxiety were assessed by locomotor activity and the amount of time spent in the central part of the arena. The OF was lit with a light intensity of 35 lx. Movement was recorded with a camera mounted above the arena and tracked with EthoVision XT, data were evaluated for time spent in the central area of the OF and distance travelled. This test, like EPM, is also based on the innate aversion of mice against bright and open spaces. The animal is considered more anxious when it spends less time in the open area of the apparatus.

### Tissue collection

The behavioural tests were followed by 5 days of vaginal smears to check oestrous cyclicity. The mice were fasted for 3–4 h before a small tail blood sample was taken to quantify serum insulin levels by ELISA (Mouse insulin 10–1247-01, Mercodia, Sweden) and glucose was measured with a blood glucose meter (Contour XT, Bayer Health Care, Mishawaka, USA). Mice were then sedated with isoflurane followed by heart puncture, and a larger blood sample for serum analyses was collected along with inguinal adipose tissue, gonadal adipose tissue, and ovaries. In a subset of mice, cerebrospinal fluid (CSF) was collected by puncturing the cisterna magna using a glass capillary with a fine tip and gentle suction^64^. Samples with suspected blood contamination were immediately discarded. Total adiponectin was analyzed in serum (diluted 1:50 000) and CSF (diluted 1:100) using an adiponectin ELISA (80,569, CrystalChem, Zaandam, Netherlands) with a sensitivity of 0.008 ng/mL, with an intra-assay coefficient variation (CV)<10%.

AMH was measured in the serum using AMH ELISA kit (AL-115, AnshLabs). The absorbance was measured on a plate reader (SpectraMax I3x, Molecular Devices, San Jose, CA, USA). Serum concentrations of sex hormones were determined with a validated liquid chromatography/mass spectroscopy (GC–MS/MS)^65^.Values below lowest level of quantification were excluded from summary calculations.

### Ovary histology

Ovaries were collected and immediately fixed in 4% paraformaldehyde for 24–48 h. They were transferred to 70% ethanol and dehydrated with different concentrations of ethanol and embedded in paraffin. Later, the ovaries were sectioned at a thickness of 5 *μ * m in a microtome. Tissues were deparaffinized with xylene and then rehydrated in graded ethanol baths (100%, 90%, 80%, 70% and 50%) followed by rinses in distilled water. Finally, they were stained with haematoxylin & eosin. The number of preantral and antral follicles, and corpus luteum were counted under a microscope^62^.

### Statistical analyses

SPSS 27.0 was used for statistical analyses. Normality of data distribution was assessed using the Shapiro–Wilk test. The data from the clinical cohort were not normally distributed in, therefore, the Mann–Whitney *U* test was used to compare women with and without PCOS. Age-adjusted analyses were performed using ANCOVA. Correlations between adiponectin, mental health scores, and free testosterone were assessed using Spearman’s rank correlation.

Data from the mouse cohort were mainly normally distributed. Our primary hypothesis was that adiponectin deficiency (APNhet) would exacerbate the effects of PNA. To test this hypothesis, two prespecified planned comparisons were performed: wt vehicle versus wt PNA to establish the effect of PNA, and wt PNA versus APNhet PNA to determine whether adiponectin deficiency had an additive effect on the PNA phenotype. An unpaired Student’s t-test was used for these two comparisons on behavioural measures, AMH levels, and ovarian morphology. Metabolic variables and sex hormone concentrations were not normally distributed and were therefore analyzed using the Mann–Whitney *U* test to answer the same biological questions. Because the study used a 2 × 2 factorial design (PNA × APNhet), a two-way analysis of variance (ANOVA) was additionally performed to assess the independent main effects of PNA and genotype, as well as their interaction. This statistical approach allowed us to address both the prespecified biological hypotheses and the overall main and interaction effects of PNA and genotype. Several sex hormone measurements were below the lower limit of quantification, therefore hormone detectability above the quantification threshold was compared between groups using Fisher’s exact test. Data are expressed as mean ± SEM, and *P* < 0.05 was considered statistically significant.

## Supplementary Information

Supplementary Information.

## Data Availability

The data supporting the findings of this study are available on request with the corresponding author.
