# Supplementary material for: Hypoadiponectinemia does not enhance anxiety-like behaviour in a lean PCOS-like mouse model
Source: Sci Rep. 2026 Sep 3;16:27688. doi: 10.1038/s41598-026-69143-9 (PMC13542225; doi:10.1038/s41598-026-69143-9)
Supplement: Supplementary file 1 [file 41598_2026_69143_MOESM1_ESM.pdf]

Supplement table 1. Characteristics of studies included in analyses.

Study	1) Kataoka et al.[22]	2) Jedel et al.[20]	3) Benrick et al.[19]	4) Johansson et al.[21]
Year	2011-2016	2005-2008	2011-2013	2009-2010
Registered at clinical trials.gov	NCT01319162	NCT00484705	NCT01457209	NCT00921492
Study design	Case-control study	Case-control study	Case-control study	Baseline measures of a randomized controlled trial
Number (PCOS/non-PCOS)	246 (63/183)	60 (30/30)	42 (21/21)	32 (32/0)
Age	18-50	18-37	18-38	18-38
BMI	PCOS: 39.9 ±4.7<br>Non-PCOS: 39.6 ±4.3	PCOS: 24.8 (18.2-40.3)<br>Non-PCOS: 24.7 (19.3-41.6)	PCOS: 31.6 ±4.12<br>Non-PCOS: 30.41 ±3.62	PCOS: 24.7 ±3.1<br>Non-PCOS: 23.2 ±3.6
Location	Sweden	Sweden	Sweden	Sweden
Recruitment	Obesity unit, Sahlgrenska university hospital	Community	Community	Community
Diagnostic criteria	NIH<br>(>35 days cycle or <8 menstruations/year and clinical (FG>6) or biochemical hyperandrogenism (T >2.1nmol/L or fT >0.035 nmol/L or FAI >5)	Rotterdam<br>2/3 criteria of:<br>Oligo-/amenorré (35 days cycle or <6 menstruations/year or amenorrhea)<br>Polycystic ovaries (>12 follicles of 2-9 mm and/or ovarian volume of > 10 ml in one or both ovaries<br>Clinical hyperandrogenism (acne or hirsutism or FG-score>8)	Rotterdam<br>2/3 criteria of:<br>Oligo-/amenorré (35 days cycle or <6 menstruations/year or amenorrhea)<br>Polycystic ovaries (>12 follicles of 2-9 mm and/or ovarian volume of > 10 ml in one or both ovaries<br>Clinical hyperandrogenism (acne or hirsutism or FG-score>8)	Rotterdam<br>2/3 criteria of:<br>Oligo-/amenorré (35 days cycle or <6 menstruations/year or amenorrhea)<br>Polycystic ovaries (>12 follicles of 2-9 mm and/or ovarian volume of > 10 ml in one or both ovaries<br>Clinical hyperandrogenism (acne or hirsutism or FG-score>8)
Exclusion criteria	Pregnancy, breastfeeding last six months, language barrier.	Any pharmacological treatment last 12 weeks, breastfeeding last 24 weeks, self-reported physical or psychiatric disease, language barrier	Pharmacological treatment last 3 months, acupuncture last 2 months, breastfeeding last 6 months, diabetes type-1, cardiovascular disease, daily smoking/ alcohol consumption	BMI<30, pharmacological treatments last 3 months, breastfeeding/acupuncture last 24 weeks, cardiovascular disease, diabetes, language barrier

Supplement table 2. Open field behavior measurements in 4-month-old wild-type (wt) and adiponectin heterozygous (APNhet) female mice with and without prenatal androgenization (PNA).

Measurements	wt veh	wt PNA	APNhet veh	APNhet PNA
Distance moved (cm)	7801 ± 850	9901 ± 664	8309 ± 617	8985 ± 892
Time in center (%)	5.43 ± 0.836	5.41 ± 0.634	5.95 ± 0.866	8.20 ± 1.49
Entries in center (n)	23.1 ± 3.34	31.5 ± 3.68	31.1 ± 3.39	34.3 ± 6.4

The effect of PNA in wt mice was analysed by an unpaired Student's t-test, and wt PNA mice were compared to APNhet PNA to determine if there was an additive effect of adiponectin deficiency. Data are expressed as mean ± SEM All comparisons were non-significant.

Supplement table 3. The main effect of prenatal androgenization (PNA exposure) and adiponectin heterozygous (APNhet) genotype, as well as the interactions on behavioural measurements in 4-month-old wild-type (wt) and APNhet female mice with and without PNA.

Measure	Effect	*F*	*P-value*
**Elevated plus maze**			
**Time in open arms (%)**	PNA	0.15	0.699
	APNhet	0.07	0.791
	PNA × APNhet	2.56	0.116
**Time in closed arms (%)**	PNA	0.97	0.330
	APNhet	0.20	0.661
	PNA × APNhet	3.43	0.070
**Time in center point (%)**	PNA	5.68	**0.021**
	APNhet	0.20	0.661
	PNA × APNhet	1.88	0.176
**Entries in closed arms (n)**	PNA	8.63	**0.005**
	APNhet	2.15	0.149
	PNA × APNhet	0.08	0.773
**Open field**			
**Distance moved (cm)**	PNA	2.96	0.092
	APNhet	0.05	0.818
	PNA × APNhet	0.73	0.399
**Time in center (%)**	PNA	0.59	0.448
	APNhet	1.48	0.231
	PNA × APNhet	0.44	0.512
**Entries in center (n)**	PNA	1.12	0.296
	APNhet	1.47	0.232
	PNA × APNhet	0.23	0.636

The main effect of PNA and APNhet, as well as the interactions (PNA x APNhet) were measured using two-way ANOVA (df = 1, 48).
